# 2-Chloro-*N*-(4-nitro­benzo­yl)benzene­sulfonamide

**DOI:** 10.1107/S1600536811009913

**Published:** 2011-03-19

**Authors:** P. A. Suchetan, Sabine Foro, B. Thimme Gowda

**Affiliations:** a Department of Chemistry, Mangalore University, Mangalagangotri 574 199, Mangalore, India; b Institute of Materials Science, Darmstadt University of Technology, Petersenstrasse 23, D-64287 Darmstadt, Germany

## Abstract

In the title compound, C_13_H_9_ClN_2_O_5_S, the N—H bond is *trans* to the C=O bond. The dihedral angle between the two aromatic rings is 85.4 (1)°. In the crystal, mol­ecules are linked into zigzag *C*(4) chains along the *b* axis through N—H⋯O hydrogen bonds.

## Related literature

For our study of the effect of substituents on the structures of *N*-(ar­yl)-amides, see: Gowda *et al.* (2000 ▶), of *N*-(ar­yl)-methane­sulfonamides, see: Gowda *et al.* (2007 ▶) and of *N*-(*p*-substituted-benzo­yl)-*p*-substituted-benzene­sulfonamides, see: Gowda *et al.* (2010 ▶); Suchetan *et al.* (2011 ▶).
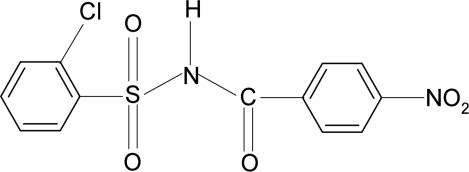


## Experimental

### 

#### Crystal data

C_13_H_9_ClN_2_O_5_S
*M* *_r_* = 340.73
Monoclinic, 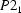

*a* = 11.097 (2) Å
*b* = 5.3063 (7) Å
*c* = 12.319 (2) Å
β = 104.24 (2)°
*V* = 703.10 (19) Å^3^
*Z* = 2
Mo *K*α radiation
μ = 0.45 mm^−1^
*T* = 293 K
0.48 × 0.16 × 0.12 mm

#### Data collection

Oxford Diffraction Xcalibur diffractometer with a Sapphire CCD detector
Absorption correction: multi-scan (*CrysAlis RED*; Oxford Diffraction, 2009 ▶) *T*_min_ = 0.815, *T*_max_ = 0.949
2641 measured reflections
2213 independent reflections
2072 reflections with *I* > 2σ(*I*)
*R*_int_ = 0.016

#### Refinement

*R*[*F* ^2^ > 2σ(*F* ^2^)] = 0.033
*wR*(*F* ^2^) = 0.087
*S* = 1.06
2213 reflections
202 parameters
2 restraints
H atoms treated by a mixture of independent and constrained refinement
Δρ_max_ = 0.23 e Å^−3^
Δρ_min_ = −0.38 e Å^−3^
Absolute structure: Flack (1983 ▶), 607 Friedel pairs
Flack parameter: 0.05 (8)

### 

Data collection: *CrysAlis CCD* (Oxford Diffraction, 2009 ▶); cell refinement: *CrysAlis RED* (Oxford Diffraction, 2009 ▶); data reduction: *CrysAlis RED*; program(s) used to solve structure: *SHELXS97* (Sheldrick, 2008 ▶); program(s) used to refine structure: *SHELXL97* (Sheldrick, 2008 ▶); molecular graphics: *PLATON* (Spek, 2009 ▶); software used to prepare material for publication: *SHELXL97*.

## Supplementary Material

Crystal structure: contains datablocks I, global. DOI: 10.1107/S1600536811009913/bt5494sup1.cif

Structure factors: contains datablocks I. DOI: 10.1107/S1600536811009913/bt5494Isup2.hkl

Additional supplementary materials:  crystallographic information; 3D view; checkCIF report

## Figures and Tables

**Table 1:** Hydrogen-bond geometry (Å, °)

*D*—H⋯*A*	*D*—H	H⋯*A*	*D*⋯*A*	*D*—H⋯*A*
N1—H1*N*⋯O1^i^	0.85 (2)	2.11 (2)	2.941 (3)	168 (3)

Symmetry code: (i) 
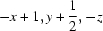
.

**Fig. 1.**
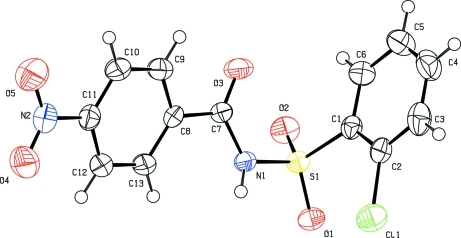
Molecular structure of the title compound, showing the atom- labelling scheme. Displacement ellipsoids are drawn at the 50% probability level.

**Fig. 2.**
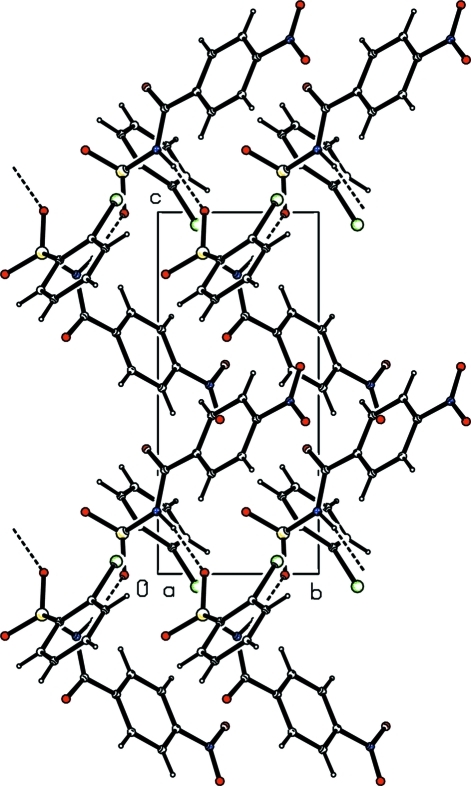
Molecular packing in the title compound. Hydrogen bonds are shown as dashed lines.

## supplementary crystallographic information

### Comment

The amide and sulfonamide moieties are important constituents of many biologically significant compounds. As a part of studying the effect of substituents on the structures of this class of compounds (Gowda *et al.*, 2000, 2007, 2010; Suchetan *et al.*, 2011), the structure of 2-chloro-*N*-(4-nitrobenzoyl)-benzenesulfonamide (I) has been determined (Fig. 1). The conformation of the N—C bond in the C—SO_2_—NH—C(O) segment has *gauche* torsions with respect to the S═O bonds. Further, the N—H bond in the C—SO_2_—NH—C(O) segment is *anti* to the C=O bond, similar to those observed in 2-chloro-*N*-(4-methylbenzoyl)-benzenesulfonamide (II) (Gowda *et al.*, 2010) and 2-methyl-*N*-(4-nitrobenzoyl)- benzenesulfonamide (III)(Suchetan *et al.*, 2011).

The molecules are twisted at the *S* atoms with the C—S(O_2_)—NH—C(O) torsional angle of 61.7 (3)°, compared to the values of 60.4 (3)° in (II) and 61.8 (5)° in (III).

The dihedral angle between the sulfonyl benzene ring and the —SO_2_—NH—C—O segment is 86.7 (1)°, compared to the values of 89.4 (1)° in (II) and 86.8 (2)° in (III).

The dihedral angle between the sulfonyl and the benzoyl benzene rings is 85.4 (1)°, compared to the values of 89.1 (2)° in (II) and 83.8 (2)° in (III).

The packing of molecules in the crystal linked by of N—H···O hydrogen bonds (Table 1) is shown in Fig. 2.

### Experimental

The title compound was prepared by refluxing a mixture of 4-nitrobenzoic acid, 2-chlorobenzenesulfonamide and phosphorous oxychloride for 3 hr on a water bath. The resultant mixture was cooled and poured into ice cold water. The solid obtained was filtered, washed thoroughly with water and then dissolved in sodium bicarbonate solution. The compound was later reprecipitated by acidifying the filtered solution with dilute HCl. It was filtered, dried and recrystallized.

Rod like colourless single crystals of the title compound used in X-ray diffraction studies were obtained by slow evaporation of its toluene solution at room temperature.

### Refinement

The H atom of the NH group was located in a difference map and later restrained to N—H = 0.86 (2) %A. The other H atoms were positioned with idealized geometry using a riding model with the aromatic C—H distance = 0.93 Å. All H atoms were refined with isotropic displacement parameters  set to 1.2 times of the *U*_eq_ of the parent atom.

### Crystal data

**Table:**

C_13_H_9_ClN_2_O_5_S	*F*(000) = 348
*M**_r_* = 340.73	*D*_x_ = 1.609 Mg m^−^^3^
Monoclinic, *P*2_1_	Mo *K*α radiation, λ = 0.71073 Å
Hall symbol: P 2yb	Cell parameters from 1433 reflections
*a* = 11.097 (2) Å	θ = 2.9–27.8°
*b* = 5.3063 (7) Å	µ = 0.45 mm^−^^1^
*c* = 12.319 (2) Å	*T* = 293 K
β = 104.24 (2)°	Rod, colourless
*V* = 703.10 (19) Å^3^	0.48 × 0.16 × 0.12 mm
*Z* = 2	

### Data collection

**Table:**

Oxford Diffraction Xcalibur diffractometer with a Sapphire CCD detector	2213 independent reflections
Radiation source: fine-focus sealed tube	2072 reflections with *I* > 2σ(*I*)
graphite	*R*_int_ = 0.016
Rotation method data acquisition using ω and φ scans	θ_max_ = 26.4°, θ_min_ = 2.8°
Absorption correction: multi-scan (*CrysAlis RED*; Oxford Diffraction, 2009)	*h* = −13→8
*T*_min_ = 0.815, *T*_max_ = 0.949	*k* = −5→6
2641 measured reflections	*l* = −15→15

### Refinement

**Table:**

Refinement on *F*^2^	Secondary atom site location: difference Fourier map
Least-squares matrix: full	Hydrogen site location: inferred from neighbouring sites
*R*[*F*^2^ > 2σ(*F*^2^)] = 0.033	H atoms treated by a mixture of independent and constrained refinement
*wR*(*F*^2^) = 0.087	*w* = 1/[σ^2^(*F*_o_^2^) + (0.052*P*)^2^ + 0.2456*P*] where *P* = (*F*_o_^2^ + 2*F*_c_^2^)/3
*S* = 1.06	(Δ/σ)_max_ < 0.001
2213 reflections	Δρ_max_ = 0.23 e Å^−^^3^
202 parameters	Δρ_min_ = −0.38 e Å^−^^3^
2 restraints	Absolute structure: Flack (1983), 607 Friedel pairs
Primary atom site location: structure-invariant direct methods	Flack parameter: 0.05 (8)

### Special details

**Table:**

Experimental. CrysAlis RED (Oxford Diffraction, 2009) Empirical absorption correction using spherical harmonics, implemented in SCALE3 ABSPACK scaling algorithm.
Geometry. All e.s.d.'s (except the e.s.d. in the dihedral angle between two l.s. planes) are estimated using the full covariance matrix. The cell e.s.d.'s are taken into account individually in the estimation of e.s.d.'s in distances, angles and torsion angles; correlations between e.s.d.'s in cell parameters are only used when they are defined by crystal symmetry. An approximate (isotropic) treatment of cell e.s.d.'s is used for estimating e.s.d.'s involving l.s. planes.
Refinement. Refinement of *F*^2^ against ALL reflections. The weighted *R*-factor *wR* and goodness of fit *S* are based on *F*^2^, conventional *R*-factors *R* are based on *F*, with *F* set to zero for negative *F*^2^. The threshold expression of *F*^2^ > σ(*F*^2^) is used only for calculating *R*-factors(gt) *etc*. and is not relevant to the choice of reflections for refinement. *R*-factors based on *F*^2^ are statistically about twice as large as those based on *F*, and *R*- factors based on ALL data will be even larger.

### Fractional atomic coordinates and isotropic or equivalent isotropic displacement parameters (Å^2^)

**Table:**

	*x*	*y*	*z*	*U*_iso_*/*U*_eq_	
Cl1	0.24217 (8)	0.24133 (17)	−0.03446 (7)	0.0518 (2)	
S1	0.40651 (6)	−0.22075 (13)	0.11243 (5)	0.02944 (16)	
O1	0.40724 (17)	−0.2097 (5)	−0.00329 (14)	0.0383 (5)	
O2	0.4343 (2)	−0.4512 (4)	0.17156 (18)	0.0423 (5)	
O3	0.4791 (2)	−0.0713 (5)	0.34556 (16)	0.0495 (6)	
O4	0.9366 (2)	0.9181 (5)	0.4214 (2)	0.0607 (7)	
O5	0.8756 (3)	0.8856 (6)	0.5731 (2)	0.0675 (8)	
N1	0.5096 (2)	−0.0071 (5)	0.17282 (18)	0.0300 (5)	
H1N	0.528 (3)	0.097 (5)	0.127 (2)	0.036*	
N2	0.8742 (2)	0.8214 (5)	0.4779 (2)	0.0435 (6)	
C1	0.2624 (2)	−0.1120 (5)	0.1320 (2)	0.0308 (6)	
C2	0.1961 (3)	0.0885 (6)	0.0729 (2)	0.0352 (6)	
C3	0.0884 (3)	0.1720 (7)	0.0994 (3)	0.0497 (8)	
H3	0.0442	0.3070	0.0608	0.060*	
C4	0.0468 (3)	0.0537 (9)	0.1834 (3)	0.0551 (10)	
H4	−0.0251	0.1111	0.2013	0.066*	
C5	0.1098 (3)	−0.1457 (8)	0.2402 (3)	0.0543 (10)	
H5	0.0800	−0.2257	0.2955	0.065*	
C6	0.2182 (3)	−0.2287 (8)	0.2153 (2)	0.0423 (7)	
H6	0.2616	−0.3635	0.2547	0.051*	
C7	0.5325 (2)	0.0433 (6)	0.2870 (2)	0.0323 (6)	
C8	0.6273 (2)	0.2411 (6)	0.3335 (2)	0.0319 (6)	
C9	0.6257 (3)	0.3356 (7)	0.4375 (2)	0.0416 (8)	
H9	0.5698	0.2706	0.4755	0.050*	
C10	0.7064 (3)	0.5262 (7)	0.4858 (2)	0.0429 (8)	
H10	0.7047	0.5926	0.5552	0.052*	
C11	0.7893 (3)	0.6146 (6)	0.4280 (2)	0.0366 (7)	
C12	0.7948 (3)	0.5216 (7)	0.3252 (2)	0.0432 (8)	
H12	0.8525	0.5835	0.2885	0.052*	
C13	0.7121 (3)	0.3329 (7)	0.2776 (2)	0.0399 (8)	
H13	0.7137	0.2678	0.2079	0.048*	

### Atomic displacement parameters (Å^2^)

**Table:**

	*U*^11^	*U*^22^	*U*^33^	*U*^12^	*U*^13^	*U*^23^
Cl1	0.0584 (5)	0.0462 (5)	0.0490 (4)	0.0055 (4)	0.0095 (3)	0.0178 (4)
S1	0.0344 (3)	0.0269 (3)	0.0271 (3)	0.0019 (3)	0.0077 (2)	−0.0002 (3)
O1	0.0459 (11)	0.0410 (12)	0.0292 (9)	0.0005 (11)	0.0119 (8)	−0.0070 (10)
O2	0.0515 (13)	0.0307 (12)	0.0448 (12)	0.0056 (10)	0.0118 (10)	0.0043 (10)
O3	0.0589 (14)	0.0627 (17)	0.0287 (10)	−0.0198 (12)	0.0145 (9)	−0.0008 (11)
O4	0.0569 (15)	0.0591 (17)	0.0662 (16)	−0.0189 (13)	0.0157 (13)	−0.0034 (14)
O5	0.0776 (18)	0.069 (2)	0.0533 (15)	−0.0174 (16)	0.0117 (13)	−0.0253 (14)
N1	0.0318 (12)	0.0338 (14)	0.0246 (11)	−0.0045 (10)	0.0071 (9)	0.0005 (10)
N2	0.0382 (13)	0.0413 (17)	0.0470 (14)	0.0011 (12)	0.0025 (11)	−0.0015 (13)
C1	0.0294 (13)	0.0312 (15)	0.0301 (13)	−0.0006 (12)	0.0040 (10)	−0.0053 (12)
C2	0.0326 (14)	0.0354 (16)	0.0343 (14)	−0.0034 (12)	0.0019 (11)	−0.0037 (13)
C3	0.0350 (16)	0.050 (2)	0.059 (2)	0.0079 (15)	0.0018 (14)	−0.0061 (17)
C4	0.0352 (17)	0.073 (3)	0.059 (2)	0.0004 (18)	0.0166 (15)	−0.016 (2)
C5	0.0476 (19)	0.071 (3)	0.0496 (18)	−0.0089 (18)	0.0217 (15)	−0.0027 (18)
C6	0.0429 (15)	0.0456 (17)	0.0392 (14)	−0.0023 (17)	0.0119 (11)	0.0049 (18)
C7	0.0304 (14)	0.0413 (17)	0.0253 (12)	0.0017 (13)	0.0071 (10)	0.0036 (12)
C8	0.0270 (12)	0.0413 (18)	0.0244 (11)	0.0015 (13)	0.0007 (9)	0.0043 (13)
C9	0.0421 (16)	0.053 (2)	0.0318 (13)	−0.0083 (14)	0.0129 (12)	−0.0023 (14)
C10	0.0453 (18)	0.055 (2)	0.0290 (14)	−0.0057 (16)	0.0106 (12)	−0.0090 (15)
C11	0.0304 (14)	0.0410 (18)	0.0353 (15)	0.0030 (13)	0.0024 (11)	−0.0017 (13)
C12	0.0347 (16)	0.061 (2)	0.0340 (14)	−0.0082 (15)	0.0083 (12)	0.0007 (16)
C13	0.0363 (14)	0.058 (2)	0.0269 (12)	−0.0073 (14)	0.0100 (11)	−0.0067 (14)

### Geometric parameters (Å, °)

**Table:**

Cl1—C2	1.732 (3)	C4—C5	1.363 (5)
S1—O2	1.418 (2)	C4—H4	0.9300
S1—O1	1.4287 (18)	C5—C6	1.384 (4)
S1—N1	1.652 (2)	C5—H5	0.9300
S1—C1	1.771 (3)	C6—H6	0.9300
O3—C7	1.205 (3)	C7—C8	1.497 (4)
O4—N2	1.210 (3)	C8—C9	1.381 (4)
O5—N2	1.219 (3)	C8—C13	1.383 (4)
N1—C7	1.392 (3)	C9—C10	1.385 (4)
N1—H1N	0.849 (18)	C9—H9	0.9300
N2—C11	1.478 (4)	C10—C11	1.376 (4)
C1—C6	1.387 (4)	C10—H10	0.9300
C1—C2	1.393 (4)	C11—C12	1.375 (4)
C2—C3	1.387 (4)	C12—C13	1.387 (4)
C3—C4	1.382 (5)	C12—H12	0.9300
C3—H3	0.9300	C13—H13	0.9300
			
O2—S1—O1	119.61 (14)	C6—C5—H5	120.1
O2—S1—N1	108.76 (13)	C5—C6—C1	120.5 (3)
O1—S1—N1	104.36 (12)	C5—C6—H6	119.8
O2—S1—C1	107.47 (13)	C1—C6—H6	119.8
O1—S1—C1	110.47 (12)	O3—C7—N1	120.9 (3)
N1—S1—C1	105.25 (13)	O3—C7—C8	121.9 (2)
C7—N1—S1	121.41 (19)	N1—C7—C8	117.2 (2)
C7—N1—H1N	122 (2)	C9—C8—C13	120.0 (3)
S1—N1—H1N	114 (2)	C9—C8—C7	116.2 (2)
O4—N2—O5	124.2 (3)	C13—C8—C7	123.8 (2)
O4—N2—C11	118.2 (3)	C8—C9—C10	120.6 (3)
O5—N2—C11	117.6 (3)	C8—C9—H9	119.7
C6—C1—C2	119.3 (3)	C10—C9—H9	119.7
C6—C1—S1	116.8 (2)	C11—C10—C9	118.1 (3)
C2—C1—S1	123.8 (2)	C11—C10—H10	120.9
C3—C2—C1	119.7 (3)	C9—C10—H10	120.9
C3—C2—Cl1	117.6 (3)	C12—C11—C10	122.8 (3)
C1—C2—Cl1	122.7 (2)	C12—C11—N2	118.7 (3)
C4—C3—C2	119.8 (3)	C10—C11—N2	118.5 (3)
C4—C3—H3	120.1	C11—C12—C13	118.2 (3)
C2—C3—H3	120.1	C11—C12—H12	120.9
C5—C4—C3	120.9 (3)	C13—C12—H12	120.9
C5—C4—H4	119.5	C8—C13—C12	120.3 (3)
C3—C4—H4	119.5	C8—C13—H13	119.8
C4—C5—C6	119.8 (3)	C12—C13—H13	119.8
C4—C5—H5	120.1		
			
O2—S1—N1—C7	−53.2 (3)	S1—N1—C7—O3	1.3 (4)
O1—S1—N1—C7	178.0 (2)	S1—N1—C7—C8	−179.8 (2)
C1—S1—N1—C7	61.7 (2)	O3—C7—C8—C9	−17.6 (4)
O2—S1—C1—C6	13.3 (3)	N1—C7—C8—C9	163.4 (3)
O1—S1—C1—C6	145.4 (2)	O3—C7—C8—C13	163.5 (3)
N1—S1—C1—C6	−102.5 (2)	N1—C7—C8—C13	−15.4 (4)
O2—S1—C1—C2	−169.9 (2)	C13—C8—C9—C10	1.5 (5)
O1—S1—C1—C2	−37.8 (3)	C7—C8—C9—C10	−177.4 (3)
N1—S1—C1—C2	74.3 (3)	C8—C9—C10—C11	−1.1 (5)
C6—C1—C2—C3	1.3 (4)	C9—C10—C11—C12	0.0 (5)
S1—C1—C2—C3	−175.5 (2)	C9—C10—C11—N2	178.9 (3)
C6—C1—C2—Cl1	−178.5 (2)	O4—N2—C11—C12	7.4 (4)
S1—C1—C2—Cl1	4.7 (4)	O5—N2—C11—C12	−173.6 (3)
C1—C2—C3—C4	−0.7 (5)	O4—N2—C11—C10	−171.6 (3)
Cl1—C2—C3—C4	179.1 (3)	O5—N2—C11—C10	7.5 (4)
C2—C3—C4—C5	−0.5 (6)	C10—C11—C12—C13	0.8 (5)
C3—C4—C5—C6	1.3 (6)	N2—C11—C12—C13	−178.1 (3)
C4—C5—C6—C1	−0.7 (5)	C9—C8—C13—C12	−0.7 (5)
C2—C1—C6—C5	−0.5 (4)	C7—C8—C13—C12	178.2 (3)
S1—C1—C6—C5	176.4 (3)	C11—C12—C13—C8	−0.5 (5)

### Hydrogen-bond geometry (Å, °)

**Table:**

*D*—H···*A*	*D*—H	H···*A*	*D*···*A*	*D*—H···*A*
N1—H1N···O1^i^	0.85 (2)	2.11 (2)	2.941 (3)	168 (3)

Symmetry codes: (i) −*x*+1, *y*+1/2, −*z*.
